# The Current State of Clinical Application of Serum Biomarkers for Canine Lymphoma

**DOI:** 10.3389/fvets.2016.00087

**Published:** 2016-09-30

**Authors:** Jeffrey N. Bryan

**Affiliations:** ^1^Comparative Oncology Radiobiology and Epigenetics Laboratory, Department of Veterinary Medicine and Surgery, University of Missouri, Columbia, MO, USA

**Keywords:** lymphoma, biomarker, serum, c-reactive protein, thymidine kinase 1, dog

## Abstract

Serum biomarkers of canine lymphoma activity for diagnosis, prognosis, and therapy monitoring have been of clinical interest for more than a decade. Tumor products, biochemical enzymes, cytokines, metabolic profiling, leakage enzymes, as well as serum proteins have been studied as biomarkers for lymphoma. Multiple biomarkers combined have been shown to be most sensitive and specific. C-reactive protein, thymidine kinase 1, and haptoglobin have been most extensively studied and commercialized in diagnostic tests, the TK Canine Cancer Panel and the Canine Lymphoma Blood Test. These tests have been evaluated either in cohorts of diseased and healthy dogs or in prospective studies of ill dogs, respectively, for application to clinical decision-making. Some evidence exists for application of these tests, but large-scale studies are lacking in a broad range of lymphoma forms. These biomarkers are commonly elevated at diagnosis and at relapse. Further study is necessary to determine if early intervention guided by biomarker elevation will improve quantity or quality of life for dogs with lymphoma.

## Introduction

Serum biomarkers of lymphoma activity for diagnosis, prognosis, and therapy monitoring have been of clinical interest for more than a decade. There are opportunities to evaluate levels of natural serum constituents, tumor produced enzymes, or even nucleic acids released from tumors that may represent dysregulated tumor drivers. Such markers offer the challenge of sample stability for shipping from remote sites, accurate and precise measurement of the diagnostic target, cross-reactivity of reagents in translation from human assays, sensitivity for the presence of lymphoma, and specificity for lymphoma as a clinical disease entity distinct from other similar diseases. While the initial diagnosis requires tissue sampling, for clinical monitoring application, robust biomarkers must be associated with clinically relevant endpoints of definitive diagnosis separate from look-alike diseases, clinical remission with greater sensitivity for residual disease than manual palpation or other clinical evaluations, or progression of disease prior to clinically detectable evidence of that event.

Many targets have been investigated as serum biomarkers for canine lymphoma since the year 2000. Tumor products alpha-fetoprotein (AFP) and microRNAs (1, 2); biochemical enzymes corticosteroid-induced alkaline phosphatase (ALP) and lactate dehydrogenase (LDH) (3, 4); cytokine tumor necrosis factor-α (TNF-α) (5); metabolic profiling by gas chromatography (6); leakage enzymes thymidine kinase 1 (TK1) and high-mobility group B1 proteins (HMGB1) (7, 8); and fucosylated serum proteins as well as serum proteins serum amyloid A (SAA), C-reactive protein (CRP), and haptoglobin (9–13) have been evaluated. Targets were generally selected as tumor-specific products or as participants in important disease-related pathways. Of the above candidates, only TK1, CRP, and haptoglobin have resulted in a commercially available biomarker test.

The focus of this review will be a brief examination of the recent biomarker candidates represented in the literature, the peer-reviewed support for application of those biomarkers discussing primarily TK1, CRP, and haptoglobin, and the evidence for clinical decision-making support published for these molecules. Most candidates have been evaluated for diagnosis and prognosis of lymphoma at the time of initial diagnosis. A few have been evaluated for monitoring remission as well. Application of biomarker monitoring to the heterogeneous disease of lymphoma, which varies by immunophenotype, histotype, anatomic location, and clinical aggressiveness, has been challenging. To date, large-scale clinical trials that would be necessary to define the role of biomarker evaluation in particular forms of lymphoma have not been conducted. The clinician is then left to evaluate the existing literature to determine appropriate clinical application of these tests.

## Tumor Products

The tumor products AFP and microRNAs have been evaluated as potential diagnostic and prognostic biomarkers for lymphoma (1, 2). Synthesized in fetal tissue and neoplastic adult tissues, AFP elevations have been reported in dogs with hepatocellular carcinoma and inflammatory/infiltrative diseases of the liver (14). Of the infiltrative diseases, some of the highest AFP levels were identified in dogs with hepatic lymphoma, which was then evaluated subsequently (1). When 63 dogs with lymphoma were compared to 80 normal controls, high-stage lymphoma dogs (27 stage III, 15 stage IV, and 9 stage V) and male dogs had the highest average AFP serum levels (1). Over the course of chemotherapy, AFP levels were lower while the dogs were in remission. Prognostic value of this biomarker for survival was not evaluated. Serum levels of a panel of microRNAs were evaluated in 61 dogs with lymphoma (41 high-grade and 20 low-grade) (2). From the panel studied, levels of four miRNAs were reduced and one increased in the serum of dogs with lymphoma relative to healthy controls (2). Only two of the microRNAs distinguished high- vs. low-grade lymphoma, and few distinguished among anatomic locations of the disease (2). No attempt was made to apply the results to prognosis based on case outcome. While each of these approaches yielded statistically meaningful differences between lymphoma and healthy dogs, neither test has been used to distinguish ill dogs from dogs with lymphoma, and neither is currently commercially available as a diagnostic test.

## Biochemical Enzymes

The serum enzymes corticosteroid-induced ALP and LDH have been evaluated as biomarkers for lymphoma (3, 4). The enzyme ALP is derived from several potential sources in the body with the corticosteroid-induced isoform being present in ill dogs or following the endogenous production or exogenous administration of corticosteroids. Measurement of ALP in 62 dogs with lymphoma did not help predict response rate or duration of remission (3). The enzyme LDH is useful for prognosis of lymphoma in humans (3). It catalyzes the final step of glycolysis, an active metabolic pathway in canine lymphoma (4). In an evaluation of 128 dogs with cancers compared to 211 ill dogs and 128 healthy dogs, LDH levels were elevated in dogs with cancers, but the overlap was significant (4). Mean levels of LDH were highest in dogs with lymphoma among the examined cancers but did not distinguish among stage of lymphoma or help predict outcome (4). The conclusion of studies of both enzymes was that they were not useful clinical tools at this stage of development.

## Cytokines and Metabolic Products

The cytokine TNF-α (5) and metabolic profiling by gas chromatography (6) have been evaluated as biomarkers of lymphoma. Serum TNF-α was studied because the cytokine has been reported to be produced by malignant lymphoblasts. However, only 3 of 25 dogs with lymphoma evaluated had detectable serum levels of the cytokine (5). Of the three with detectable levels, all became undetectable following therapy. However, the low frequency of detection led to the conclusion that TNF-α has limited value as a biomarker for lymphoma (5). Lymphoma is well known to alter body metabolism, so the serum metabolic profile of 21 dogs with lymphoma was compared to 13 healthy dogs using gas chromatography (6). Using gas chromatography–mass spectrometry analysis, 29 metabolites were used to differentiate between dogs with lymphoma and healthy controls with 16 metabolites having significantly different levels (6). Of three cases analyzed that entered complete remission after treatment, all three had metabolic profiles typical of the healthy dogs (6). The authors concluded that this metabolic approach could yield biomarkers of utility in monitoring lymphoma, but the analysis is not widely available for this application.

## Cellular Leakage Enzymes

Candidate cellular leakage enzymes evaluated as biomarkers include HMGB1 and TK1 (7, 8). The HMGB1 enzyme is generally an intracellular molecule that is released by damaged or dying cells; it can also be secreted by activated monocytes, macrophages, and astrocytes (8). A study of 16 dogs with lymphoma showed significantly elevated HMGB1 levels at diagnosis compared to normal dogs, although the ranges overlapped somewhat (8). Through the course of chemotherapy, HMGB1 levels diminished concurrent with the degree of perceived remission of disease. One dog with a partial remission retained higher levels than the dogs in complete remission (8). The authors concluded that because of this phenomenon, the enzyme may have value as a prognostic marker, but measurement is not currently available as a diagnostic test. Thymidine kinase 1 (TK1) is a cytoplasmic enzyme that catalyzes the pyrimidine salvage pathway and is often expressed in neoplastic cells (7, 15). First reported in dogs in 2004, serum TK1 has been evaluated as a tool for both prognosis and disease monitoring of lymphoma (7). Utilizing a radio-enzyme assay, serum samples from 21 healthy beagles, 8 juvenile beagles, and 65 dogs with lymphoma were evaluated for TK1 activity. The mean TK1 activity in the juvenile dogs was found to be higher than the mean TK1 activity of the healthy adult dogs (7). Serum TK1 activity in dogs with lymphoma was significantly higher than that of normal dogs or dogs with inflammation or other tumors (7). Evaluation with ROC analysis yielded a cut-point with a true positive rate of 92% and a false positive rate of only 1.9% (7). Further, serum TK1 activity was significantly higher in high-stage (≥stage 3 disease) than in low-stage lymphoma patients (7). Pretreatment serum TK1 activity above an arbitrary cut-point was prognostic, with high TK1 portending a much shorter survival, likely related to stage (7). A subsequent report confirmed that the early results could be recapitulated in a different population using a non-radiometric immunoassay with high correlation (16). This assay has shown promise as a biomarker of disease, prognosis, and monitoring (13, 17–19). The commercial application of this assay will be discussed subsequently.

## Serum Proteins

Measurement of fucosylated serum proteins as well as SAA, CRP, and haptoglobin has been evaluated as biomarkers of lymphoma disease and treatment monitoring (9–13). A proteomic approach to analysis of glycosylation of serum proteins identified significant changes from normal in levels of 109 fucosylated peptides derived from serum proteins (11). Levels of 54 of these modified peptides changed from pretreatment to posttreatment measurement as well (11). Levels were seen to change at relapse, but the timing was not reported relative to clinical detection. The technique was concluded to offer potential for disease monitoring but has not been developed commercially. The acute phase protein SAA was elevated in dogs with lymphoma, decreased with therapy, but was not seen to elevate at relapse (10). The authors concluded that SAA was not a useful marker of relapse in dogs with lymphoma.

C-reactive protein has been evaluated by several groups as a biomarker of lymphoma activity. As a sole biomarker, serum CRP has been shown to be elevated at diagnosis in most dogs with lymphoma compared to healthy dogs, and to decrease to a range indistinguishable from healthy dogs when in remission (9, 20). The conclusion by the authors of both studies reported here was that CRP is not a useful biomarker for remission monitoring by itself. To that end, CRP has been evaluated in combination with another acute phase protein haptoglobin. A study of 16 dogs identified elevations of CRP, haptoglobin, or both in all 16 dogs (21). Elevations of CRP were generally of greater magnitude than those of haptoglobin. High-grade and advanced stages (most of the cases) had greater magnitude elevations. The authors were unable to evaluate the relationship of biomarker level to stage or prognosis because of the homogeneity of the cases in the study. Using a surface-enhanced laser desorption ionization time of flight (SELDI-TOF) protein detection method, another group identified CRP and haptoglobin as potential biomarkers of lymphoma and created ELISA-based assays for those proteins (12, 22). A bioinformatics approach was used to construct a model based on the two biomarkers to identify lymphoma cases to calculate a canine lymphoma blood test (cLBT) value (23). Evaluating 35 lymphoma samples and 34 control samples from non-lymphoma diseased dogs, a sensitivity of 91%, specificity of 88%, and accuracy of 89.9% was calculated for prediction at diagnosis (22). In a subsequent set of 96 dogs evaluated prospectively with careful clinical follow-up, the test performed similarly with a sensitivity of 75%, specificity of 91%, and accuracy of 85% in initial diagnosis samples where 32 of the dogs were ultimately confirmed to have lymphoma (22). One caveat to these findings is that the reported values were calculated using 158 samples from 96 dogs, so independence of the samples in the calculation may confound the result. The same group evaluated 57 dogs serially to determine the utility of this biomarker pair and algorithm in monitoring remission status and predicting relapse. Comparing clinical remission status to cLBT value, dogs in complete remission had significantly lower values than dogs at diagnosis or in partial remission or with progressive disease (12). Serum cLBT values at diagnosis and lowest value measured during therapy were associated with survival, with lowest values performing better (12). Falling serum cLBT levels lagged behind lymph node palpation for defining remission and rose prior to nodes enlarging in the dogs studied (12). Finally, serum CRP has also been evaluated in conjunction with TK1 with a calculated Neoplasia Index (NI) value resulting (13). Among 83 dogs with lymphoma evaluated, the NI was significantly elevated compared to controls (13). Dogs with T cell lymphoma had generally lower levels of TK1 (13). In this study, 16 of 156 control dogs had an NI in the middle to upper range, which would not be distinguishable from lymphoma (13). Follow-up of these dogs for monitoring of disease development was not reported.

## Commercialization

Currently, two tests are available commercially as serum biomarkers for lymphoma in dogs, the cLBT (Avacta Animal Health, Wetherby, UK) and the TK1 Canine Cancer Panel (NI) (VDI Laboratory, Simi Valley, CA, USA). Both laboratories have locations in the US for convenient shipping, and both offer clinically useful turnaround time of results, typically within 1–3 days. Head-to-head comparison of the two testing methods for diagnostic support, prognostication, and remission monitoring has not been reported.

The cLBT has the advantage of literature that describes its use in the setting of initial diagnosis, prognostication, and remission monitoring (12, 22). Importantly, the computational background of the calculation of cLBT values has also been reported (23). Publication of this algorithm allows clinicians utilizing this test to assess the methods behind the analysis and provides a description of the ongoing machine learning built into the system (23). The cLBT was evaluated prospectively in cohorts of clinically ill dogs with performance similar to the unblinded comparison groups reported early in development of the test (12, 23). Prospective evaluation of cases in this manner is critical to understanding the real performance of such biomarker tests. Finally, the proprietors of the cLBT have reported prognostic data from prospectively collected cases as well, offering some understanding of the likely performance of the test (12). What is as yet unclear about the cLBT is how it performs across grades of lymphoma, whether the early detection of loss of remission will alter outcome for dogs with lymphoma, and whether the prognostic value of the test will hold up in larger populations of dogs with lymphoma.

The NI calculation has the advantage of including not only CRP as a measure of systemic disease but also TK1, which is tumor-specific. Both these biomarkers are well described in literature but primarily evaluated in known cases (13, 17). The algorithm calculating the NI is only generally described and is specifically referred to as proprietary (13, 24). It is worth noting that in one study of the NI in clinically healthy dogs, two dogs were prospectively identified as having elevated NI, which subsequently died of lymphoma (24). Overall, this study reported a sensitivity of the NI in dogs without disease for future disease development was 82% and the specificity 91%. The NI could differentiate dogs developing cancer within 6 months with an ROC AUC of 0.93. This study reported significant differences in the ranges of CRP, TK1, and NI between dogs without cancer and those that developed cancer. However, the graphical representation of these ranges recorded a large number (>50) of values of both CRP and TK1 that were considered outliers (24). The methods did not describe whether these were included in the statistical comparison, but the box and whisker plot does not appear to reflect that they were (24). Each dog in the study that died of cancer had an elevated NI.

When considering whether to use a serum biomarker in lymphoma decision-making, a clinician must consider the state of knowledge of that test. The currently available commercial tests are relatively well described in the literature. The cLBT has been assessed prospectively in dogs of unknown initial diagnosis with similar disease presentations (12). The cLBT has also been reported to have prognostic significance in these prospective evaluations. What currently lacks for this test is an understanding of whether low-grade lymphomas have similarly elevated scores to high-grade lymphomas, whether prognosis of test score at the beginning and end of chemotherapy will remain prognostic in the broad patient population, and whether identifying early evidence of loss of remission before clinical node enlargement present will extend comfort or survival for these dogs. The NI calculation has the advantage of testing in a relatively broad range of cancers and a reasonable number of dogs with lymphoma. It also utilizes a biomarker that is a cytosolic leakage enzyme that may be more tumor-specific than only acute phase proteins. However, it has not yet been prospectively tested in a population of dogs with both lymphoma and similar disease presentations to understand how well it discriminates. It also appears that among a large number of apparently healthy dogs (*n* = 360), a relatively large proportion will have biomarker values higher than most of the population raising the possibility of false positives (24). Although both TK1 and the NI track with remission status, it is not yet clear whether there is prognostic significance to the score, although level of TK1 itself has been shown to be prognostic (7). As with any biomarker test, identification of early loss of remission is of unknown clinical benefit in dogs at this time.

## Conclusion

Low-cost serum biomarkers for lymphoma diagnosis, prognosis, and disease monitoring are appealing clinical tools. The current literature contains some support for use of biomarkers in each of these applications. Commercially available tests are relatively sensitive to the presence of high-grade lymphoma in the body. No currently evaluated approaches have successfully separated indolent from aggressive lymphomas or immunophenotype of lymphoma. Utilizing genomic and epigenomic features may someday fill this gap. Further study is necessary to fully define the roles and accuracy of serum biomarker in the diagnosis and management of canine lymphoma.

## Author Contributions

Dr. JB performed the literature search and wrote the entire article.

## Conflict of Interest Statement

Dr. JB has previously been a paid consultant and paid lecturer for the Avacta Company regarding the Canine Lymphoma Blood Test. The nature of this consultancy was a focus group on application of blood biomarker tests and a lecture to practitioners on lymphoma monitoring.
